# Equine Chorionic Gonadotropin Modulates the Expression of Genes Related to the Structure and Function of the Bovine Corpus Luteum

**DOI:** 10.1371/journal.pone.0164089

**Published:** 2016-10-06

**Authors:** Liza Margareth Medeiros de Carvalho Sousa, Gabriela Pacheco Mendes, Danila Barreiro Campos, Pietro Sampaio Baruselli, Paula de Carvalho Papa

**Affiliations:** 1 Department of Surgery, School of Veterinary Medicine and Animal Science, University of São Paulo, São Paulo, São Paulo, Brazil; 2 Department of Veterinary Sciences, Faculty of Veterinary Medicine, Federal University of Paraíba, Areia, Paraíba, Brazil; 3 Department of Animal Reproduction, School of Veterinary Medicine and Animal Science, University of São Paulo, São Paulo, São Paulo, Brazil; Max Delbruck Centrum fur Molekulare Medizin Berlin Buch, GERMANY

## Abstract

We hypothesized that stimulatory and superovulatory treatments, using equine chorionic gonadotropin (eCG), modulate the expression of genes related to insulin, cellular modelling and angiogenesis signaling pathways in the bovine corpus luteum (CL). Therefore, we investigated: 1—the effect of these treatments on circulating insulin and somatomedin C concentrations and on gene and protein expression of INSR, IGF1 and IGFR1, as well as other insulin signaling molecules; 2—the effects of eCG on gene and protein expression of INSR, IGF1, GLUT4 and NFKB1A in bovine luteal cells; and 3—the effect of stimulatory and superovulatory treatments on gene and protein expression of ANG, ANGPT1, NOS2, ADM, PRSS2, MMP9 and PLAU. Serum insulin did not differ among groups (P = 0.96). However, serum somatomedin C levels were higher in both stimulated and superovulated groups compared to the control (P = 0.01). In stimulated cows, lower expression of INSR mRNA and higher expression of NFKB1A mRNA and IGF1 protein were observed. In superovulated cows, lower INSR mRNA expression, but higher INSR protein expression and higher IGF1, IGFR1 and NFKB1A gene and protein expression were observed. Expression of angiogenesis and cellular modelling pathway-related factors were as follows: ANGPT1 and PLAU protein expression were higher and MMP9 gene and protein expression were lower in stimulated animals. In superovulated cows, ANGPT1 mRNA expression was higher and ANG mRNA expression was lower. PRSS2 gene and protein expression were lower in both stimulated and superovulated animals related to the control. In vitro, eCG stimulated luteal cells P4 production as well as INSR and GLUT4 protein expression. In summary, our results suggest that superovulatory treatment induced ovarian proliferative changes accompanied by increased expression of genes providing the CL more energy substrate, whereas stimulatory treatment increased lipogenic activity, angiogenesis and plasticity of the extracellular matrix (ECM).

## Introduction

The use of equine chorionic gonadotropin (eCG) in cattle follicular stimulation and superovulation has substantial effects on follicular development [1] and corpus luteum (CL) function [2]. It stimulates the growth of the dominant follicle [3, 4] and promotes the increase of circulating progesterone (P4) concentrations in the subsequently estrus cycle [5, 6]. These findings were associated to augmented CL diameter and to the stimulation of P4 production by the CL itself [2, 7]. Additionally, it was observed that stimulatory treatment with eCG provoked morphophysiological changes in the CL such as the increase of density and volume of small and large luteal cells and the increase in the mitochondrial density [8]. In the molecular level, the impacts of eCG have not been totally elucidated yet. A previous study performed by our research group analyzed by microarray the effects of stimulatory and superovulatory treatments, both using eCG, on luteal gene expression profile of recipient and donor cows, respectively. It has been observed that eCG causes changes in the expression of multiple genes, particularly those related to P4synthesis, metabolism, cell differentiation, proliferation and angiogenesis [9]. Based on these previous results, we hypothesized that eCG, associated to these hormone protocols, modulates the expression of insulin signaling pathways compounds, promoting an increase of glucose availability to bovine luteal cells. Insulin, in turn, will act synergistically with eCG, potentiating its role to promote luteinization and favor the augment of P4 synthesis.

Therefore, the primary aim of the experiments was to investigate the effects of stimulatory and superovulatory treatments, using eCG, on circulating insulin and somatomedin C concentrations and on gene and protein expression of insulin receptor (INSR), insulin-like growth factor 1(IGF1) and its receptor, IGFR1, as well as other signaling molecules such as solute carrier family 2 (facilitated glucose transporter), member 4 (*SLC2A4*) and nuclear factor of kappa light polypeptide gene enhancer in B-cells (NFKB1A), which encode the glucose transporter type 4 (GLUT4) and nuclear factor kappa-B P50 subunit (P50) proteins, respectively. The second aim was to determinate the pathways by which eCG exerts its effects on the expression of the aforementioned insulin-related molecules in bovine luteal cells.

Luteinization and CL development mean increase in blood flow and constant remodeling [10]. Additionally, remodeling of the connective tissue and tissue degradation are associated with maintenance of the function and the regression of CL [11, 12]. In bovine granulosa cells, the expression of some of these factors increased after FSH and IGF1 stimulation [13], which leads us to infer that these factors can be in several ways related to insulin signaling and that stimulatory and superovulatory treatments with eCG modulate their expression. Thus, the third aim of this study was to investigate the expression of angiogenin, ribonuclease, RNase A family, 5 (ANG) and angiopoietin 1 (ANGPT1), which are involved in the initiation and establishment of angiogenesis [14, 15] and the expression of factors involved in cell modeling as nitric oxide synthase 2, inducible (NOS2), adrenomedullin (ADM), protease, serine, 2 (trypsin 2) (PRSS2), matrix metallopeptidase 9 (MMP9) and plasminogen activator, urokinase (PLAU) in the CL from synchronized (control), stimulated and superovulated cows.

## Material and Methods

### Ethics Statement

All procedures were performed according to ethical principles approved by the Ethics Committee for the Use of Animals at the School of Veterinary Medicine and Animal Sciences, University of São Paulo, Brazil (Protocol numbers: 2715/2012 and 3153/2013).

### Animals

Hormonal treatments were performed in the Department of Animal Reproduction, School of Veterinary Medicine and Animal Science, at the Campus of Pirassununga–São Paulo. Seventeen crossbred non-lactating, multiparous Nellore (*Bos indicus*) cows aged between 2–5 years, with approximately 600 Kg and body condition score between 2–3, from a scale of 1 to 5 [16] were used. These cows were maintained on pasture supplemented with concentrate and mineral salt (17.03% cornmeal, 4.65% soybean meal, 1.13% urea, 0.11% ammonium sulfate, 1.69% minerals, 0.53% salt and 74.86% corn silage) and fresh water *ad libitum*.

### Estrus synchronization and ovulation

The animals were randomly divided into control (n = 5), stimulated (n = 6) and superovulated groups (n = 6) and submitted to the synchronization of follicular wave and ovulation using a P4 device-based protocol [9]. Briefly, on day 0 (random day of the estrous cycle), all animals received an intravaginal P4 device (1g, Primer, Technopec, Brazil) and an intramuscular injection of 2 mg estradiol benzoate (Estrogin, Farmavet, Brazil). On day 8, P4 devices were removed from control and stimulated cows, and 0.150 mg of d-cloprostenol (prostaglandin [PG] F2α, Prolise, Arsa, Argentina) were administered. Stimulated cows also received 400 UI of eCG (Novormon, Syntex, Argentina). After 48 h, on day 10, 0.025 mg of gonadotropin-released hormone (GnRH, Gestran Plus, Arsa, Argentina) were administered in both control and stimulated cows. Superovulated animals received 2000 UI of eCG on day 4 and 0.150 mg of PGF2α on day 6 after P4 device insertion. On day 7, P4 device was removed and 0.150 mg PGF2α was administered again. Twelve hours later, on day 8, 0.025 mg of GnRH was administered. Seven days after GnRH administration, i.e. six days after ovulation, the CL of all groups were collected after slaughter, frozen in liquid nitrogen and stored at -80°C for later RNA and protein extraction.

### Serum insulin and somatomedin C determinations

Blood samples were also obtained after slaughter using 50 ml sterile tubes. All tubes were centrifuged at 3,000 x g for 10 min and the obtained serum was stored at -20°C until the analysis of insulin and somatomedin C concentrations. At the end of the experiments, all samples from each cow were analyzed in the same assay in duplicate using commercial RIA kits (Insulin Coat-a-Count^®^, Siemens Medical Solutions Diagnostics, USA and IGF1 RIA kit, A15729, Beckman Coulter Diagnostics, USA). The standard curve for insulin ranged from 2 to 200 μU ml^-1^. The sensibility was 90% and the intra-assay coefficient of variation (CV) was 6.95%. Likewise, 500 μl of samples were analyzed for somatomedin C. The standard curve ranged from 82.09 to 582 ng ml^-1^. The sensibility was 70% and the intra-assay CV was 3.77%.

### Microarray analysis, identification and selection of candidate genes

To identify the molecular changes on the CL from cows stimulated and superovulated with eCG, microarray analysis was done previously [9]. According to these analyses, 242 transcripts were up regulated and 111 down regulated in the stimulated group, whereas 111 transcripts were up regulated and 113 down regulated in the superovulated, compared to the control (fold change of ± 1.5; P < 0.05). A database was generated (access number GEO: GSE37844), from which we identified and selected differentially expressed cell modelling-, angiogenesis- and insulin pathway-related genes (Table 1). Although they do not appear on the list, gene and protein expression of IGF1, IGFR1 and GLUT4 were also analyzed, since they are important compounds of the insulin system [17]. Finally, differentially expressed factors were subjected to validations.

**Table 1:** Microarray results for genes validated by real time PCR, western blotting and immunohistochemistry.

Gene	Gene symbol	Fold change	P value	Fold change	P value
		Stimulated vs. Control		Superovulated vs. Control	
Adrenomedullin	*ADM*	1.69	0.02*	----	----
Angiogenin, ribonuclease, RNAse A family, 5	*ANG*	1.63	0.01	----	----
Angiopoietin 1	*ANGPT1*	1.83	0.009	----	----
Insulin receptor	*INSR*	----	----	-3.01	0.02
Matrix metallopeptidase 9	*MMP9*	-2.26	< 0.0001	-2.06	0.004
Nuclear factor of kappa light polypeptide gene enhancer in B-cells inhibitor, alpha	*NFKBIA*	----	----	1.64	0.03
Nitric oxide synthase 2, inducible	*NOS2*	-1.95	0.007	-1.52	0.003
Plasminogen activator, urokinase	*PLAU*	-1.61	0.01	----	----
Protease, serine, 2 (trypsin 2)	*PRSS2*	-11.6	< 0.0001	1.9	0.05
Ras homolog gene family, member Q	*RHOQ*	----	----	1.60	0.01
Src homology domain containing transforming protein 2	*SHC1*	----	----	1.58	0.001

*±1.5 fold (P ≤ 0.05)

### cDNA preparation and real time PCR

Total RNA was isolated using the trizol protocol (Life Technologies, USA) and the RNA quality was evaluated by using a NanoDrop 2000 (Thermo Fisher Scientific Inc., USA) and 2% agarose gel.

Real time PCR was carried out as previously described [18]. Briefly, total RNA (1 μg) was submitted to reverse transcription using the SuperScript III kit (Life Technologies, USA). The resulting cDNA was used in subsequently PCR reactions, which were performed with an automated fluorometer (ABIPrism^®^ 7500, Life Technologies, USA), using 96-well optical plates. Primers are described in Table 2. Common thermal cycling settings were used to amplify each transcript (2 min at 50°C, 10 min at 95°C then 40 cycles of 15 s at 95°C and 60 s at 60°C). Relative quantification was performed by normalizing the target genes signals with GADPH, β-actin (ACTB) and α-tubulin (TUBA1A) signals (as housekeeping genes). The amplification efficiency was analyzed using the ΔCT-based method (ΔΔCt) [18].

**Table 2:** List of primers used for real time (TaqMan) PCR.

Gene	Primers	Sequences	Amplicon	GenBank n°
**ADM**	Forward	5´GTAGAGACCCAGGTACTAAATCAAA 3´	92	NM_173888.3
	Reverse	5´ATTTCTTCAAGGCTGGGAAGTACTG 3´		
**ANG**	**----**	ID Bt85_m1*	164	NM_001078144.1
**ANGPT1**	**----**	ID Bt03249550327928_m1*	69	NM_001076797.1
**MMP9**	**----**	ID Bt03216000_g1*	108	NM_174744
**NOS2**	**----**	ID Bt03249599_m1*	61	NM_001070267.1
**PLAU**	**----**	ID Bt03212963_m1*	78	NM_174147.2
**PRSS2**	**----**	ID Bt03224030_m1*	129	NM_174690.1
**INSR**	Forward	5’GCTGCTGCCTGGGAATTA 3’	68	AY574999.1
	Reverse	5’CCATCTGGCTGCCTCTTT 3’		
**IGF1**	Forward	5’TTGCACTTCAGAAGCAATGG 3’	91	NM_001077828.1
	Reverse	5’GAAGAGATGCGAGGAGGATG 3’		
**IGF1R**	Forward	5’ CCTCATCAGCTTCACCGTCTACT 3’	70	NM_001244612.1
	Reverse	5’GCGTCCTGCCCGTCATACT 3’		
**SHC1**	Forward	5’ GTGAGGTCTGGGGAGAAGC 3’	127	NM_001164061.1
	Reverse	5’ GGTTCGGACAAAGGATCACC 3’		
**RAC2**	Forward	5’ TGAGATGGCCTCGGTCATT 3’	78	NM_175792.2
	Reverse	5’ TGTTGGTCGCTAACAGAAGCA 3’		
**RHOQ**	Forward	5’TGCTTCTCCGTGGTAAATCC 3’	122	NM_001205498.1
	Reverse	5’CCTTGTTCCACACAGACAGG3’		
**NFKB1A**	Forward	5’ACCACTTATGACGGGACTACAC 3’	127	DQ464067.2
	Reverse	5’CGCCGAAACTGTCCGAGAAA 3’		
**SLC2A4**	Forward	5´TGGGAGCCACGCTGCCTGCCTGTGGGGCA 3´	116	NM_174604.1
	Reverse	5´CTGGGAAGGAAGAGGGCCATGCTGT 3´		
**ACTB**	Forward	5’TCATCACCATCGGCAATGAG 3’	141	NM_173979.3
	Reverse	5’CATCGTACTCCTGCTTGCTGA 3’		
**TUBA1A**	Forward	5’ TGTTCGCTCAGGTCCTTTTGG 3’	52	BT_0323101
	Reverse	5’ CCCTTGGCCCAGTTGTTG 3’		
**GAPDH**	Forward	5´GCGATACTCACTCTTCTACCTTCGA 3´	140	NM_001034034.2
	Reverse	5´TCGTACCAGGAAATGAGCTTGAC 3´		

*Pre designed assay from Applied Biosystems (Life Technologies, USA).

### Western blotting

For total protein extraction, CL samples were homogenized in NET-2 lysis buffer (50 mM Tris-HCl [pH 7.4], 300 mM NaCl and 0.05% NP-40) containing 1 μl/ml protease inhibitor cocktail (Sigma-Aldrich, St. Louis, MO, USA) using a Polytron (Brinkmann Instruments, Westbury, NY, USA) and centrifuged at 10,000 x g for 10 min at 4°C. The supernatant was used as total cellular extract. For nuclear protein extraction, CL samples were firstly pulverized in liquid nitrogen as previously described [19]. These samples were centrifuged at 1000 x g for 10 min at 4°C in PBS buffer containing DTT and PMSF, followed by further centrifugation at 15,000 x g for 30 s at 4°C in hypotonic buffer (1 M HEPES-KOH, 1 M MgCl_2_, 2 M KCl, 200 mM DTT, 0.2 M PMSF and protease inhibitor). The samples were then further centrifuged at 12,000 x g for 2 min in hypertonic buffer (1 M HEPES-KOH, 1 M MgCl_2_, 0.5 M EDTA, 220 mM DTT and 0.2 M PMSF). The protein concentration was determined by the Bradford method [20].

Both whole cellular and nuclear extracts (50μg) were resolved by 8–15% sodium dodecyl sulfate-polyacrylamide gel electrophoresis (SDS-PAGE) gels, depending on the protein molecular weight, and eletrophoretically transferred onto immunoblot polyvinylidene difluoride membranes (Bio-Rad). For reference, pre-stained molecular weight standards (Kaleidoscope^™^, Bio-Rad Laboratories Inc., USA) were included on all gels. Western blot analysis was performed using the primary antibodies listed in Table 3, and peroxidase labelled anti-rabbit, anti-mouse and anti-goat at 1: 7,500 dilution as secondary antibodies (Amersham Biosciences, GE Healthcare Life Science, USA). β-actin (ACTB) was used as reference protein. Both primary and secondary antibodies were prepared in PBS containing 2.5% (v/v) non-fat milk. The blots were visualized using an Enhanced Chemiluminescence (ECL) Kit (Amersham Biosciences, GE Healthcare Life Science, USA) and images were captured by ChemiDoc MP Image system (Bio-Rad Laboratories Inc., USA).

**Table 3:** Antibodies used for Western blotting and Immunohistochemistry.

Antibodies	Isotype	Epitope	Dilution	Supplier (order n°)
**ADM**	Rabbit polyclonal IgG	Purified human ADM	1:500	Elsasser et al., 2007
**ANG**	Rabbit polyclonal IgG	101–200 human ANG protein	1:1000	Biorbyt (orb101736)
**ANGPT1**	Rabbit polyclonal IgG	N-term of human ANGPTL1	1:1000	Abbiotec (251299)
**GLUT4**	Rabbit polyclonal IgG	C-terminus	1:1000	Millipore (07–1404)
**IGF1**	Rabbit polyclonal IgG	49–118 of human IGF1	1:1000	Santa Cruz (sc-9013/H-70)
**IGF1R**	Rabbit polyclonal IgG	1350–1367 of human IGFR1	1:1000	Abcam (ab5497)
**NOS2**	Rabbit polyclonal IgG	Citokine-induced murine macrophages	1:500	Lifespam Biosciences (LS11686/37570)
**INSR**	Rabbit polyclonal IgG	C-term of human insulin Rβ	1:1000	Santa Cruz (sc-711/C-19)
**MMP9**	Rabbit polyclonal IgG	382–393 human MMP9	1:1000	Biorbyt (orb13583)
**P50**	Goat polyclonal IgG	C terminus of human NFκB p50	1:1000	Santa Cruz (sc-1190/C-19)
**PRSS2**	Rabbit polyclonal IgG	Native pancreatic tripsinogen	1:1000	MyBioSource (MBS622131)
**PLAU**	Goat polyclonal IgG	Human purified PLAU	1:500	Acris (APO22255SU-N)
**ACTB**	Mouse monoclonal IgG1	N-term of β-isoform of actin	1:10000	Sigma-Aldrich—A1978 (AC-15)

### Immunohistochemistry for MMP9, PRSS2, ANG, ANGPT1, ADM, NOS2 and PLAU

Immunoperoxidase method was used to detect MMP-9, PRSS2, NOS2, ANG, ANGPT1, ADM and PLAU in 2 μm tissue sections prepared from on CL per cow using 3 section per CL for each animal per group to assure accuracy of description as already published [21]. The primary antibody (Table 3) for each protein was diluted in PBS at the following dilutions: MMP-9 1:100; PRSS2 1:1000; NOS2 1:200; ANG 1:100; ANGPT1 1:200; ADM 1:1000; PLAU 1:300 and incubated for overnight at 4°C. Negative controls were prepared using IgG isotype control antibodies (Normal rabbit IgG for all antibodies; Santa Cruz Biotechnologies, USA). The slides were observed with an Olympus BX 50 microscope equipped with a CCD color video camera (Olympus DP71; Olympus America Inc, USA), and the images were captured using Axio Vision software (Carl Zeiss, Germany).

### Isolation of bovine luteal cells and eCG treatment

Ovaries were collected at a local abattoir and processed essentially as described [22]. The stage of the cycle was determined by macroscopic observation of the ovaries (follicles and corpora lutea) and the CL from stage II of luteal phase (days 5–10 after ovulation; [23] were used. The luteal tissue was dissociated with collagenase I (17100–017, Gibco, Life Technologies, USA). The cell viability was determined by the trypan blue exclusion method and luteal cells preparations with more than 85% viability were used. Bovine luteal cells were seeded in 96-well plates (~4 x 10^4^ cells / well) in DMEM/F12 (12400–024, Gibco, Life Technologies, USA) containing 5% fetal bovine serum (10437–028, Gibco, Life Technologies, USA) and 1% antibiotic-antimycotic solution (A5955, Sigma-Aldrich Co, USA), pH 7.2–7.4, for 24 h at 37°C in a humidified atmosphere of 5% CO_2_. Then, the medium was replaced by fresh medium supplemented with 0, 5, 25, 50, 200 or 400 UI of eCG (Novormon 5000, Syntex, Argentina) for 24 h. The concentrations of eCG were determinate based on experimental data documenting the efficacy of eCG international units in distinct cell models [24–26] and on which is preconized for cattle follicular stimulation in Brazil (400 UI; [3, 4]. For P4 determinations, the medium was collected and stored at -20°C. For RNA and protein extraction, trizol and NET-2 lysis buffer were added, respectively. Finally, the cells were scrapped, collected, snap-frozen in liquid nitrogen and stored at -80°C until real time PCR and western blotting analyses (as described above).

### Progesterone determinations

Medium P4 concentrations were determined by validated RIA (r^2^ = 0.986 and P < 0.0007), using the commercial kit Progesterone Coat-a-Count^®^ (Siemens Medical Solutions Diagnostics, USA). The standard curve ranged from 0.16 to 55.2 ng ml^-1^. The sensibility was 92% and the intra- and interassay CVs were 3.14% and 2.88%, respectively.

### Statistical analysis

All experiments for real time PCR, western blotting and hormone determinations were repeated three times. Data were examined for normality and homogeneity using the Shapiro-Wilk and Bartlett tests, respectively. Data that were not normally distributed were transformed to natural logarithms. All data are represented as mean ± SEM. Gene expression, protein expression and P4, insulin and somatomedin C concentrations were analyzed using One-Way ANOVA followed by Dunnett multiple comparison test. The statistical significance between two groups was determined with Unpaired t test, Welch corrected. Nonlinear regression analysis was used to evaluate the correlation between gene and protein expression; gene expression and hormone concentrations; and, protein expression and hormone concentrations. All analyses were performed using the GraphPad Prism 5.0 software (GraphPad Software, USA) and differences of P ≤ 0.05 were considered significant.

## Results

### Insulin and somatomedin C

**Fig 1:**
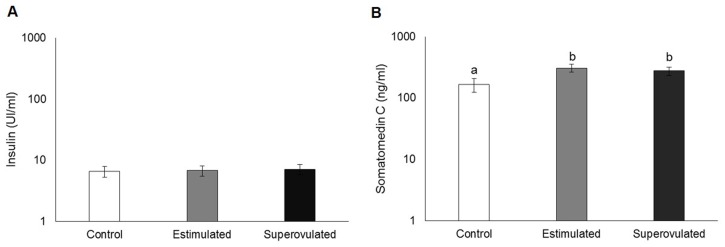
Serum profiles of insulin (A) and somatomedin C (B) in control (white bars), stimulated (gray bars) and superovulated (black bars) groups. Data are presented in a logarithm scale. Bars with different letters differ at P ≤ 0.05.

The profiles of serum insulin and somatomedin C of stimulated, superovulated and control cows are presented in Fig 1. There was no difference regarding insulin levels among groups (P > 0.05; Fig 1A). However, serum somatomedin C levels were higher in both stimulated and superovulated groups, compared to the control (only synchronized cows; P = 0.01; Fig 1B).

### Expression of INSR, IGF1, IGFR1, SLC2A4/GLUT4, NFKB1A/P50, SHC1, RAC2 and RHOQ in the CL of stimulated and superovulated cows

**Fig 2:**
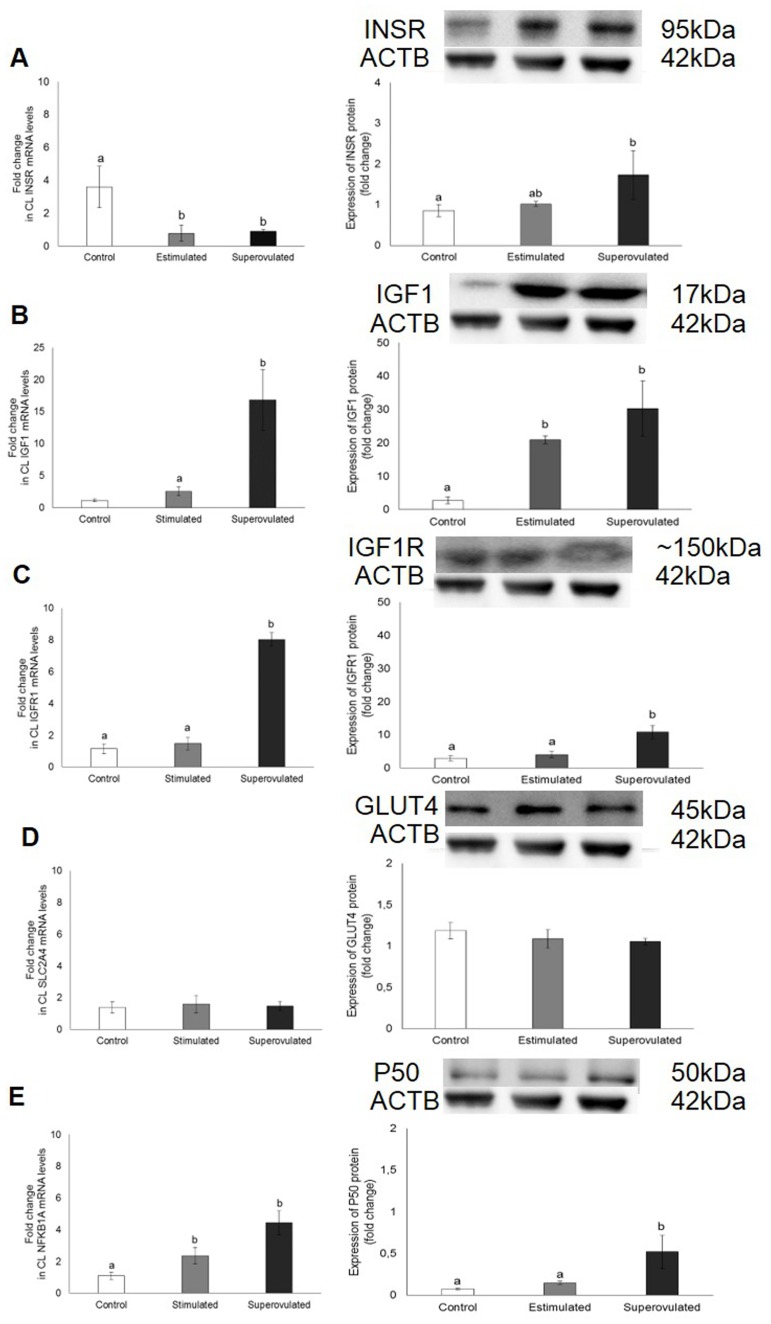
Gene (left column) and protein expression (right column) of INSR (A), IGF1 (B), IGFR1 (C), SLCA2A4/GLUT4 (D) and NFKB1A/P50 (E) in the corpus luteum from control (white bars); stimulated (gray bars) and superovulated (black bars) cows. For protein expression, 50 μg of total or nuclear protein were used. Representative blots are shown. Β-actin (ACTB; ~42 kDa) was used as reference protein. Bars with different letters differ at P ≤ 0.05.

The expression of insulin-related signaling molecules on the CL of stimulated and superovulated cows compared to the control group are demonstrated on Fig 2.

**Fig 3:**
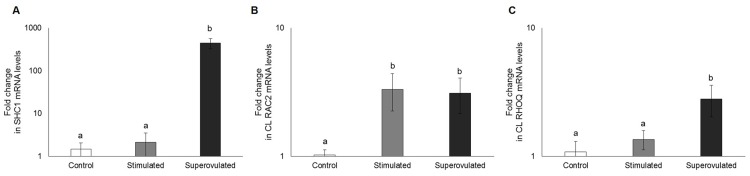
Expression of SHC1 (A), RAC2 (B) and RHOQ mRNA (C) in the corpus luteum from control (white bars), stimulated (gray bars) and superovulated (black bars) cows. Data are presented in a logarithm scale. Bars with different letters differ at P ≤ 0.05.

In stimulated animals was observed lower *INSR* mRNA expression (P = 0.007; Fig 2A), higher *NFKB1A* expression (P = 0.0003; Fig 2D) and higher IGF1 protein expression (P < 0.0001; Fig 2B) compared to the control. In superovulated cows, was observed lower *INSR* mRNA expression (P = 0.0007; Fig 2A), but higher INSR protein expression (P = 0.03; Fig 2A) and higher gene and protein expression of IGF1 (P < 0.0001; Fig 2B), IGFR1 (P < 0.0001; Fig 2C) and NFKB1A (P = 0.003 and 0.0007, respectively; Fig 2D). GLUT4 expression did not differ among the groups (P > 0.05; Fig 2E). Additionally, RAC2 mRNA expression was higher in both stimulated and superovulated groups (P = 0.02), whereas SHC1 and RHOQ mRNA levels were higher only in the superovulated group (P < 0.0001 and P = 0.03, respectively; Fig 3).

Comparing gene expression, regression analysis indicated a positive linear correlation between the expression of INSR and SLC2A4; SHC1 with RAC2 and NFKB1A; and, NFKB1A and SLC2A4 (Table 4). When comparing gene expression with protein, a positive correlation was observed only between NFKB1A and its protein P50. Additionally, negative correlation was observed between INSR mRNA expression and serum insulin levels.

**Table 4:** Regression analysis between the expression of different genes or between gene and protein expression and between a particular gene and serum insulin levels.

Variables				
Y	X	R2	P-value*	Equation
*SLC2A4*	*INSR*	0.4464	0.004	Y = 0.32x + 0.6853
*SLC2A4*	*INSR*	0.4464	0.004	Y = 0.32x + 0.6853
*IGFR1*	*IGF1*	0.9625	< 0.0001	Y = 0.235x + 1.8775
*RAC2*	*SHC1*	0.6049	0.0004	Y = 0.006x + 1.1842
*NFKB1A*		0.6544	< 0.0001	Y = 0.0059x + 1,6759
*SLC2A4*	*NFKB1A*	0.2558	0.04	Y = 0.3079x + 0.6897
P50		0.866	0.0003	Y = 0.1068x + 0.0172
*INSR*	Insulin (serum)	0.2694	0.03	Y = -0.558x + 6.3785

*P<0.05

### Effect of eCG on progesterone production by bovine luteal cells

**Fig 4:**
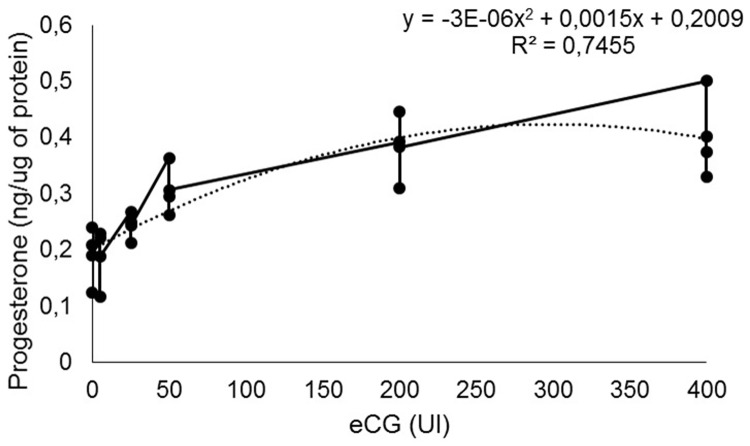
Progesterone concentrations (ng μg^-1^) of bovine luteal cells treated with different doses (UI) of eCG. P < 0.0001.

In the control group, P4 concentration was 0.19 ± 0.03 ng μg^-1^ and a positive correlation between luteal P4 production and eCG concentrations could be observed (R^2^ = 0.74; P < 0.0001; Fig 4).

### Effect of eCG on INSR, IGF1, GLUT4 and P50 expression in bovine luteal cells

**Fig 5:**
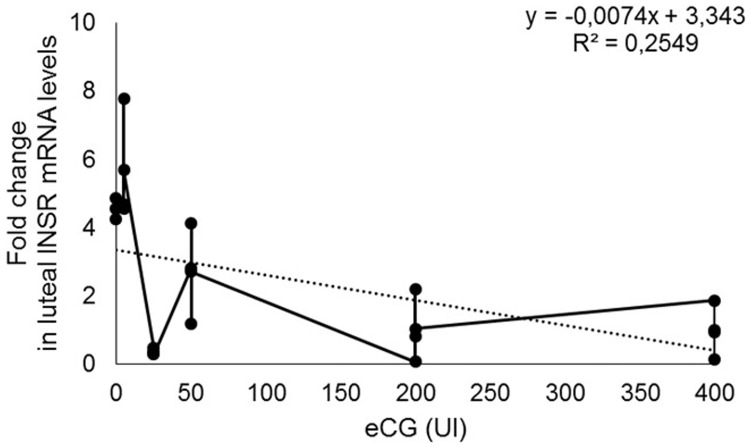
Expression of INSR mRNA (fold change) on bovine luteal cells treated with different doses of eCG (0, 5, 25, 50, 200 and 400 UI). P = 0.01.

The effects of eCG on IGF1, IGFR1, NFKB1A and SLC2A4 mRNA expression were not observed, once the results obtained by real time PCR were undetectable. The exception was INSR, which mRNA expression presented a negative correlation with eCG concentrations (R^2^ = 0.25 P = 0.01; Fig 5).

**Fig 6:**
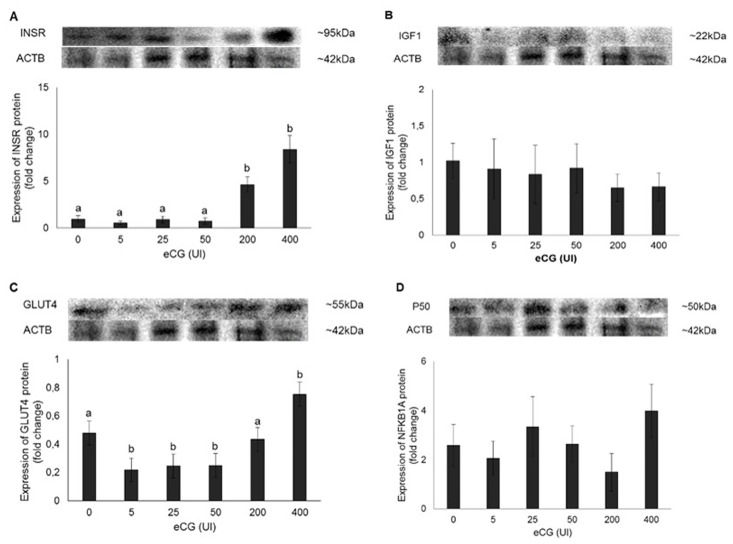
Expression of INSR (A), IGF1 (B), GLUT4 (C) and P50 (D) protein on bovine luteal cells under different eCG concentrations (0, 5, 25, 50, 200 and 400 UI). For protein expression, 50 μg of total or nuclear protein were used. Representative blots are shown. β-actin (ACTB; 42 kDa) served as reference protein. Bars with different letters differ at P ≤ 0.05.

On Fig 6, the profiles of INSR, IGF1, GLUT4 and P50 protein expression can be visualized. INSR (A) and GLUT4 (C) expression presented a positive correlation with eCG concentrations (R^2^ = 0.80 P < 0.0001 y = 0.0181x + 0.4658 and R^2^ = 0.58 P = 0.0001 y = 0.0014x + 0.2584, respectively). No effect of eCG on IGF1 (B) neither on P50 (D) expression was observed.

### Expression of ANGPT1, ANG, MMP9, PRSS2, ADM, NOS2, and PLAU in the CL of stimulated and superovulated cows

**Fig 7:**
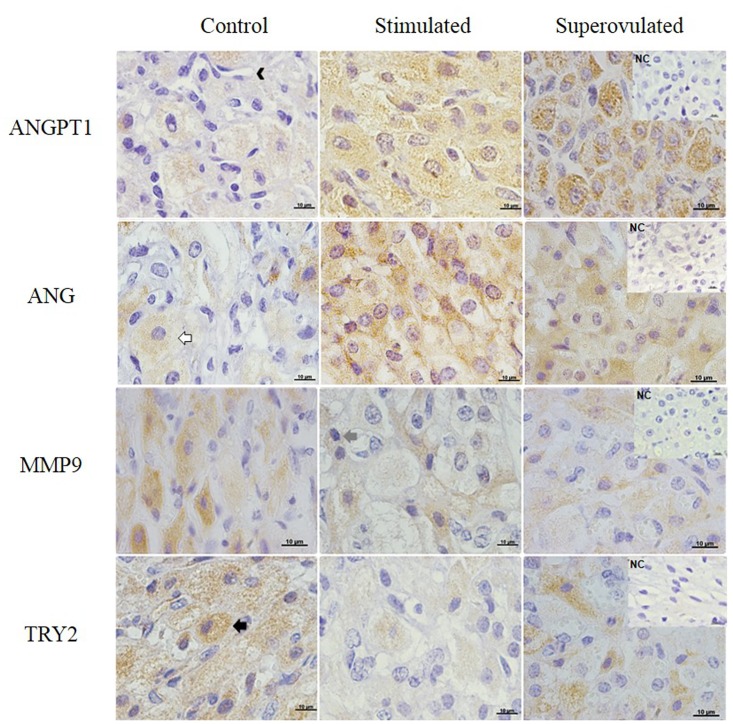
Angiopoietin-1 (ANGPT1; first row), Angiogenin (ANG; second row), MMP-9 (third row) and PRSS2 (fourth row) expression in the bovine CL detected by immunohistochemistry. Positive signals can be observed as the orange-brown color in the cytoplasm of large and small luteal cells, stroma and endothelial cells in the control (left column), stimulated (middle column) and superovulated (right column) animals. NC = negative control. Bars = 10μm.

**Fig 8:**
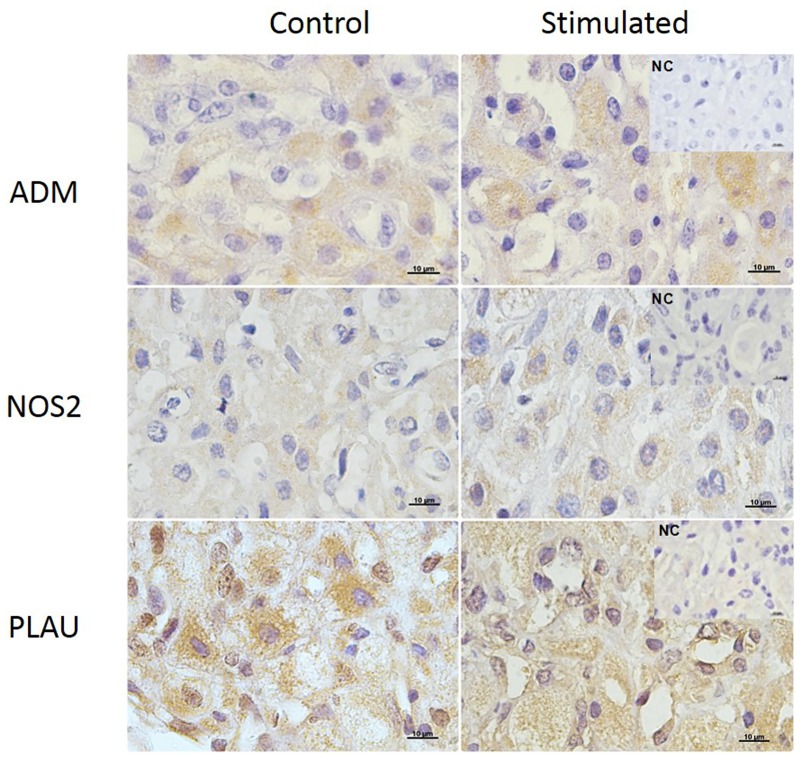
Adrenomedullin (ADM; first row), NOS2 (second row) and PLAU (third row) expression in the bovine CL detected by immunohistochemistry. Positive signals can be observed as the orange-brown color in the cytoplasm of large and small luteal cells and stroma and endothelial cells in the control (left column) and stimulated (right column) animals. NC = negative control. Bars = 10μm.

**Fig 9:**
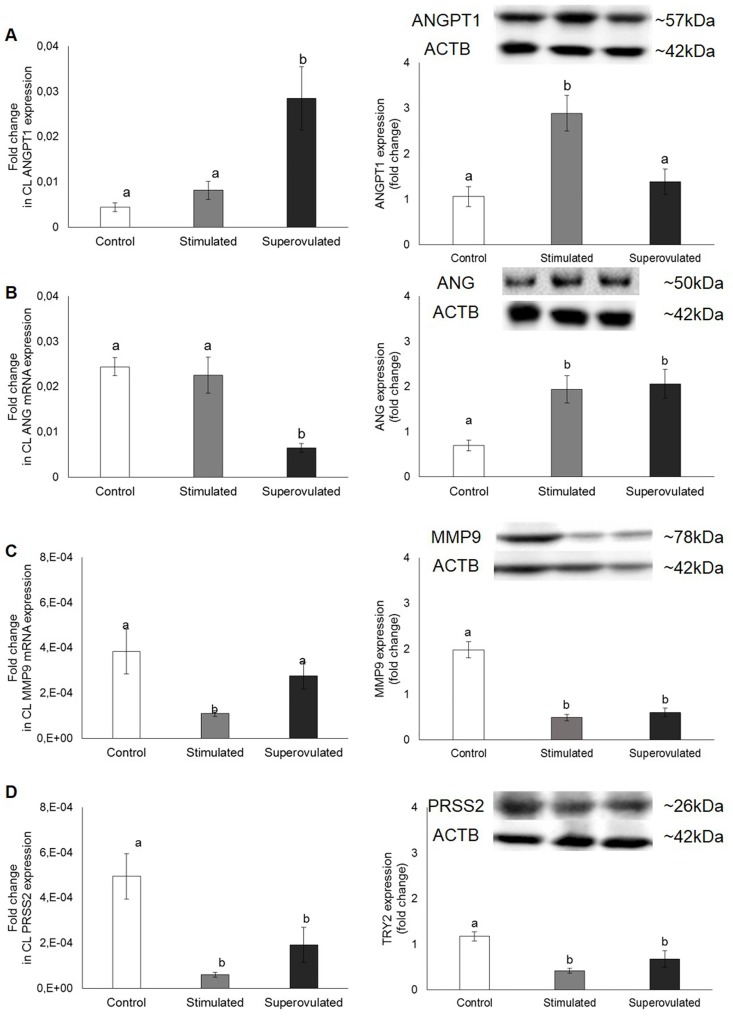
Gene (left column) and protein (right column) expression of ANGPT1 (A), ANG (B), MMP9 (C) and PRSS2 (D) in the corpus luteum from control (white bars), stimulated (gray bars) and superovulated (black bars) cows. For protein expression, 50 μg of total protein were used. Representative blots are shown. β-actin (ACTB; 42 kDa) served as reference protein. Bars with different letters differ at P ≤ 0.05.

The gene and protein expression of angiogenic and cell modelling signaling molecules on the CL of stimulated and superovulated cows compared to the control group (only synchronized cows) are demonstrated on Figs 7, 8 and 9.

Tissue localization of molecules involved in angiogenesis (ANGPT1 and ANG) and cell modelling (MMP9, PRSS2, ADM, NOS2 and PLAU) was performed by IHC. The signals of all proteins were evenly distributed within the CL and localized only in the cytoplasm of large and small luteal cells, stroma and endothelial cells (Figs 7 and 8).

Considering mRNA expression, a positive effect of cow treatment on angiopoietin gene expression could be observed (R2 = 0.50 P = 0.001). The expression of *ANGPT1* was higher in the superovulated group (P = 0.01), while in the protein level, ANGPT1 expression was higher in the stimulated group (P < 0.0001; Fig 9A). Angiogenin mRNA expression was lower in the superovulated group (P = 0.001), whereas its protein was higher in both stimulated and superovulated groups (P = 0.0004; Fig 9B). A negative correlation was observed between *ANG* expression and the treatment (R2 = 0.62 P = 0.0002), whereas a positive correlation could be observed between the treatment and ANG expression (R2 = 0.32 P = 0.02). Gene and protein expression of MMP9 were lower in the stimulated group (P = 0.03 and P < 0.0001, respectively; Fig 9C). A positive correlation between MMP9 protein expression and the treatment was observed (R2 = 0.89 P = 0.02). Trypsin 2 mRNA and protein expression were lower in both stimulated and superovulated groups (P = 0.002; Fig 9D), but no effect of treatment was observed.

**Fig 10:**
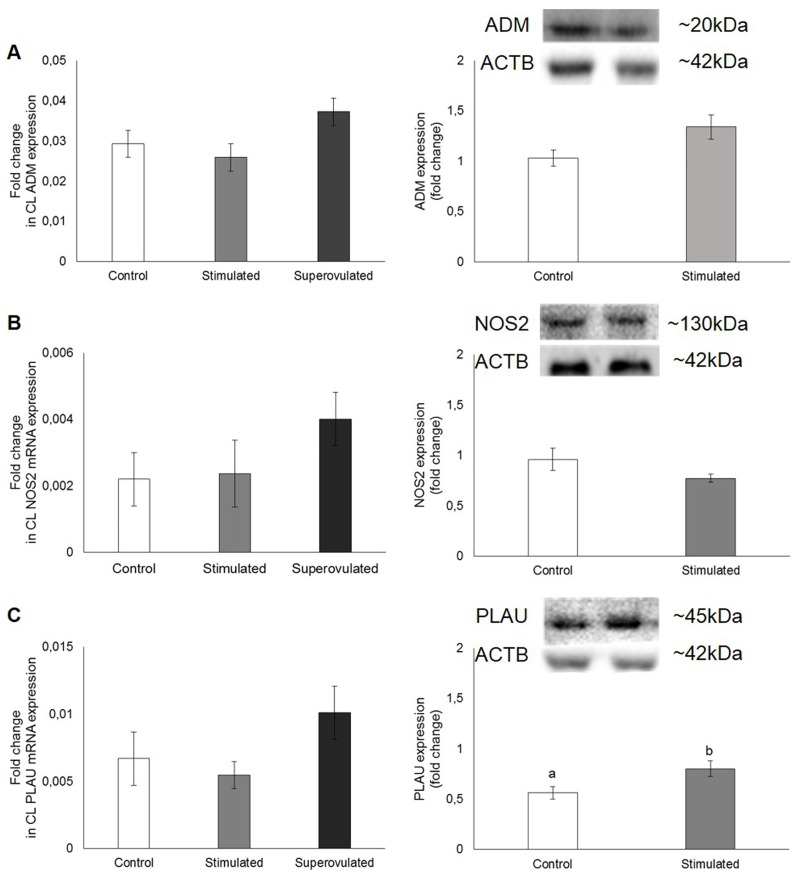
Gene (left column) and protein (right column) expression of ADM (A), NOS2 (B) and PLAU (C) in the corpus luteum from control (white bars), stimulated (gray bars) and superovulated (black bars) cows. For protein expression, 50 μg of total protein were used. Representative blots are shown. β-actin (ACTB; 42 kDa) served as reference protein. Bars with different letters differ at P ≤ 0.05.

Additionally, stimulatory treatment with eCG had no effect on ADM, NOS2 and PLAU mRNA expression in bovine CL tissue (P ≥ 0.05; Fig 10), although protein expression of PLAU was higher in the stimulated group, compared to the control (P = 0.04; Fig 10C).

Comparing gene expression with P4 levels, a negative correlation was observed between *MMP9* and *NOS2* expression and P4 (R2 = 0.33 P = 0.03 y = -4E-05x + 0.0004 and R2 = 0.42 P = 0.02 y = -0.0002x^2^ + 0.0006x + 0.0036, respectively). At the protein level, a positive correlation between P4 levels and ANGPT1 could be observed (R2 = 0.37 P = 0.02 y = 0.3351x + 0.5449).

## Discussion

Starting from the premise that eCG, associated to stimulatory and superovulatory treatments, provokes morphological changes [8] and alters the bovine corpus luteum global gene expression profile [9], we investigated the influence of these hormonal protocols on some luteal physiological aspects. Therefore, CL from synchronized cows submitted or not (control group) to eCG treatment before or after follicular deviation (superovulatory and stimulatory treatments, respectively) were collected and underwent gene and protein expression analysis of signaling molecules related to the insulin/IGF system, cell modelling, angiogenesis and steroidogenesis.

Firstly, it was observed that circulating insulin concentrations did not differ among experimental groups. However, somatomedin C concentrations increased nearly 2-folds in both stimulated and superovulated animals compared to the control. In the superovulated group, which was submitted to another protocol receiving a dose of eCG 5 times higher than the stimulated group, the increase of serum somatomedin C levels was accompanied by the increase of luteal IGF1 gene and protein expression. In the stimulated group, this increase was observed only at the protein level, which would explain the augment of peripheral levels. It is well established that IGFs are intraovarian key regulators [27], playing an important role in the interactions with respect to both hormone synthesis and survival responses [28]. Some of these actions are synergistic with gonadotropins, although most are not sustainable with IGFs alone and require gonadotropin actions, thereby designating IGFs as co-gonadotropins [27]. In mouse ovary, eCG increased IGFs expression by increasing the expression of pregnancy-associated plasma protein-A (PAPPA), which is a metalloprotease responsible for the cleavage of IGF binding protein 4 (IGFBP4) in the ovary [29], decreasing its affinity for IGFs and increasing IGF peptide bioavailability [30]. The bovine ovary also expressed PAPPA [31], becoming a possible mechanism whereby eCG induces IGF1 expression in the corpus luteum.

Insulin growth factor 1 acts primarily through type I IGF receptor [32]. Depending upon the cell type, IGF1 activates the PI3K pathway and/or the MAPK pathway [33]. In superovulated animals, it has been described an augment of the expression of INSR protein and of signaling molecules from the MAPK pathway, including *SHC1* and *RAC2*, accompanied by the increase of *NFKBIA*/P50 expression as well as from the CAP/CBL pathway, as *RHOQ* and *SLC2A4*, which expression was higher in these animals. In stimulated animals, there was a decrease of *INSR* and *SLC2A4* expression and an increase of *NFKBIA*, but changes in the protein expression were not observed. These findings suggest that not only the concentration, but also the time point when eCG is administered, influences its pharmacodynamics. When it is administered before follicular divergence, i.e. in the superovulatory treatment, whose goal is the development and maturation of a great number of follicles, eCG appears to activate, via IGF1 system, the necessary pathways to increase glucose availability and cell growth to the formation of various corpora lutea. When it is administered after follicular deviation, i.e. in the stimulatory treatment, eCG stimulates the final growth and maturation of the dominant follicle, resulting in the formation of a CL greater in volume. In addition, it stimulates the expression of steroidogenic enzymes, such as STAR, and the regulation of genes related to lipid synthesis, favoring the P4 production [9].

Using bovine luteal primary cell culture, the effects of eCG on the insulin/IGF1 system were investigated. Luteal cells were stimulated with different doses of eCG (0, 5, 25, 50, 200 and 400 UI), but regardless of eCG concentrations tested, cell viability remained similar to the control group (mean of 80%) after 24 hours. It occurred probably due to the short time in culture, which did not request extra nutritional supplementation for luteal cells to survive. Furthermore, the presence of serum in the medium provides the necessary nutrients to the cells [34]. P4 concentrations increased in a dose-dependent manner after eCG treatment, which is in accord to the literature [35–37]. In addition, P4 concentrations were accompanied by an increase of INSR and GLUT4 protein expression. GLUT4 is a glucose transporter related to insulin, playing an important role in energy balance. Until recently, it was believed that the GLUT4 was expressed exclusively by muscles and adipose tissue [38]. However, GLUT4 expression was detected in reproductive tissues such as endometrium, corpus luteum and placenta [39, 40]. On in vivo studies, the eCG, associated to stimulatory and superovulatory protocols, had no effect on INSR neither on GLUT4 protein expression. However, when administered alone (in vitro), it stimulated the expression of both proteins, suggesting a more metabolic action, once it does not affect IGF1 protein expression. Although the IGF1 and the INSR are structurally and functionality related proteins and share many of the same signaling molecules, they modulate different responses within the cell [41]. Insulin growth factor 1 has been implicated mostly in mitogenic function and INSR in metabolic actions [42]. Therefore, the above-mentioned results suggest that, depending on the conditions, the eCG priority is mitogenesis, via IGFR1, or metabolism, via INSR. The mere presence of GLUT4/*SLC2A4* in the bovine CL suggests insulin sensitivity by the luteal cells, but further studies are necessary.

Secondly, this study was designed to investigate the influence of stimulatory and superovulatory protocols with eCG on angiogenesis, cell modeling and steroidogenesis, since, in the ovary, the primary proangiogenic factors are regulated by gonadotropins, steroids, and other growth factors [43–45], as IGF1 as mentioned above. Therefore, the expression of mRNA and protein of ANGPT1, ANG, MMP9, PRSS2, ADM, NOS2 and PLAU were evaluated.

In the present study, both ANGPT1 and ANG were expressed by luteal (small and large) and endothelial cells. These findings are in agreement with previous reports, on which these proteins were described in the bovine corpus luteum [46, 47]. Corpus luteum formation and regression are related to active angiogenesis and angiolysis, respectively [48, 49], which are mechanisms that require the destabilization of blood vessels [50]. ANGPT1 maintains and stabilizes blood vessels developed by vascular endothelial growth factor–VEGF and the decrease on its gene expression appears to regulate CL vascular remodeling [51, 52]. Angiogenin, in turn, interacts with endothelial and smooth muscle cells to induce a wide range of cellular responses including cell migration, invasion, proliferation, and formation of tubular structures [53]. As shown in earlier studies [21, 54], neither stimulatory nor superovulatory protocols influenced the VEGF system mRNA and protein expression. In contrast to VEGF, ANGPT1 itself does not initiate endothelial network organization, but stabilizes networks initiated by VEGF, presumably by stimulating the interaction between endothelial and periendothelial cells. This indicates that ANGPT1 may act at later stages than VEGF [55]. On the other hand, ANG assumes an essential role in endothelial cell proliferation and serves as a crossroad in the process of angiogenesis induced by other angiogenic factors, such as VEGF [56]. It was also observed that ANGPT1 mRNA expression increased after superovulatory treatment, while its protein increased after stimulatory treatment. Angiogenin protein expression increased in both stimulated and superovulated animals. Although VEGF pathways are central mediators of angiogenesis, IGFs also play a role [55]. Insulin growth factors promote endothelial cell migration and tube formation in vitro [57]. Insulin growth factor 1 and IGF2 stimulate HIF1A expression [58], and IGF1 induces VEGF synthesis [59, 60] via HIF1-dependent and -independent pathways [61]. The direct relation between IGF1 and angiogenesis in the bovine corpus luteum is not clear yet, but these results suggest that ANGPT1 may contribute to the maintenance of vessel stability throughout early CL formation and that IGF1 may act as an angiogenic factor, especially in stimulated cows.

Adrenomedullin is a peptide highly conserved across species [62, 63] and widely expressed in various organs and tissues, including reproductive organs [64–66]. In ovary, ADM protein and its mRNA were detected in the follicles and CL of rat and human [64, 67], and the present study is the first report of the presence of this peptide in the bovine CL. The expression of ADM mRNA and the presence of its protein in the cytoplasm of luteal, endothelial and stromal cells, indicates that it is transcribed and synthesized by the CL. The hallmark biological effects of ADM are vasodilation and hypotensive effects in the vascular systems of most species [62, 68]. However, it can exerts pleiotropic actions, including cell proliferation [69, 70], migration [71, 72], apoptosis [73, 74], inflammation [75, 76], angiogenesis [77], and hormone secretion [78]. Adrenomedullin enhanced also P4 production in human granulosa luteal cells [79]. However, no association between P4 levels and ADM expression was observed in this study, perhaps due the fact that the concentrations of ADM required to increase P4 production were higher than physiological concentrations [79]. Additionally, stimulatory and superovulatory protocols with eCG did not affect ADM gene and protein expression. These findings are not consistent with earlier reports on which the maturation and luteinization of rat granulosa cells induced by gonadotropins were associated with a significant suppression in *ADM* expression [65], and that ADM suppressed eCG-stimulated P4 release in rat CL [67]. It can be explained by the fact that were different species (rat versus cattle), different cell type (granulosa versus luteal cells) and that FSH not LH induced granulosa cells differentiation and decreased ADM production [58].

Angiogenesis is a process that involves vasodilatation, vascular permeability, degradation of extracellular matrix and migration of endothelial cells. Vasodilatation involves nitric oxide [55]; proteinases of the plasminogen activator, matrix metalloproteinase (MMP) families influence angiogenesis by degrading matrix molecules and by activating or liberating growth factors (basic fibroblast growth factor—bFGF, VEGF and IGF1), sequestered within the extracellular matrix [80]. Urokinase-type plasminogen activator (PLAU) is also essential for revascularization [81]. In this study, we observed that the bovine corpus luteum expressed the mRNA and the protein of NOS2, MMP9, PRSS2 and PLAU.

Nitric oxide (NO) is a small diffusible signaling molecule regulating a diverse range of cellular processes [82, 83]. The bovine CL has two types of NOS, NOS2 and NOS3, and we observed the immunostaining of NOS2 in endothelial cells and luteal cells as previous described [84]. Neither stimulatory nor superovulatory treatments affected NOS2 expression in the bovine CL, suggesting other regulatory mechanisms. The stimulation of IGF1 has been demonstrated to induce NO production in endothelium [85], but insulin is a more potent stimulator [86]. Furthermore, the biological function of ADM in many cell types is mediated by NO induction [87]. Adrenomedullin can induces NO production by at least two known mechanisms. It can trigger the activation of phosphatidylinositol 3-kinase and protein kinase B/v-akt murine thymoma viral oncogene homolog (PI3K/AKT) signaling pathway, such as IGF1 and insulin, resulting in the phosphorylation and increased activity of NOS [88] or it can upregulate intracellular Ca^2+^ to increase the NOS activity [89]. According to Cornish and colleagues [90], there is an interaction between the mitogenic pathways activated by IGF1 and ADM in osteoblasts. In these cells, a functional IGFR1 receptor is required for adrenomedullin-stimulated phosphorylation of p42/44 MAP kinases and the adrenomedullin receptor appear to be important for the IGF1 mitogenic response [90]. However, further work is required to clarify the real importance of ADM in the bovine CL and to explore the possibility of interaction between it and IGF1 in this organ.

Proteinases such as MMP9 and PRSS2 are thought to form cascades that degrade tissue barriers and thus promote cell invasion and increased expression and secretion of these enzymes are associated with the metastatic capacity of tumor cells [91]. However, MMPs are not the only protease system involved in luteal function and regression [92]. The plasminogen activator (PA) system, which includes plasmin, tissue-type PA (tPA) or urokinase-type PA (μPA) and two specific PA inhibitors (PAI-1 and PAI-2) [92, 93], may also play a role during different luteal stages. In the present study, we observed that MMP9, PRSS2 and PLAU are regulated by hormonal protocols in CL. Both stimulatory and superovulatory treatments suppressed MMP9 and PRSS2 protein expression, while stimulatory treatment induced PLAU expression, in relation to the control. These findings are consistent with previous results, in which it was observed that gonadotropins decreased MMPs expression and increased PLAU in the monkey and human CL [94], suggesting a role of these proteases in luteal maintenance and a hormonal regulation of their action in the developing CL. Additionally, according to Kliem and collaborators [12], it is possible that in the developing CL, endothelial cells expressing PLAU become detached from the extracellular matrix (ECM) and are able to migrate into the unvasculated stroma to form new capillaries. These results indicate that in the CL of stimulated cows, there is a mobilization to promote vascularization, which is the fundamental step to enable the supply of luteal cells.

In summary, our results suggest that eCG induces IGF1 production in the corpus luteum to increase its responsiveness to gonadotropins, as was observed in dominant follicles at the time of follicle selection [95, 96]. The morphological changes induced by the superovulatory treatment in the ovaries are accompanied by increased expression of genes providing the CL more energy substrate, whereas the increase in P4 production presented by the CL after stimulatory treatment seems to be related to increased lipogenic activity, angiogenesis and plasticity of the ECM.

## Supporting Information

S1 Fig Uncropped image file of the INSR (A), IGF1 (B), IGF1R (C), GLUT4 (D), P50 (E) and ACTB (F) SDS-PGE gels shown in Fig 2. C, ST and SV = control, stimulated and superovulated cows.
(TIFF)

S2 Fig Uncropped image file of the INSR (A), IGF1 (B), GLUT4 (C), P50 (D) and ACTB (E) SDS-PGE gels shown in Fig 6. eCG concentrations (0, 5, 25, 50, 200 and 400 UI).
(TIFF)

S3 Fig Uncropped image file of the ANGPT1 (A), ANG (B), MMP9 (C), PRSS2 (D) and ACTB (E) SDS-PGE gels shown in Fig 9. C, ST and SV = control, stimulated and superovulated cows.
(TIFF)

S4 Fig Uncropped image file of the ADM (A), NOS2 (B), PLAU (C) and ACTB (D) SDS-PGE gels shown in Fig 10. C and ST = control and stimulated cows.
(TIFF)
