# Supplementary figures and images for: Equine Chorionic Gonadotropin Modulates the Expression of Genes Related to the Structure and Function of the Bovine Corpus Luteum
Source: PLoS One. 2016 Oct 6;11(10):e0164089. doi: 10.1371/journal.pone.0164089 (PMC5053489; doi:10.1371/journal.pone.0164089)

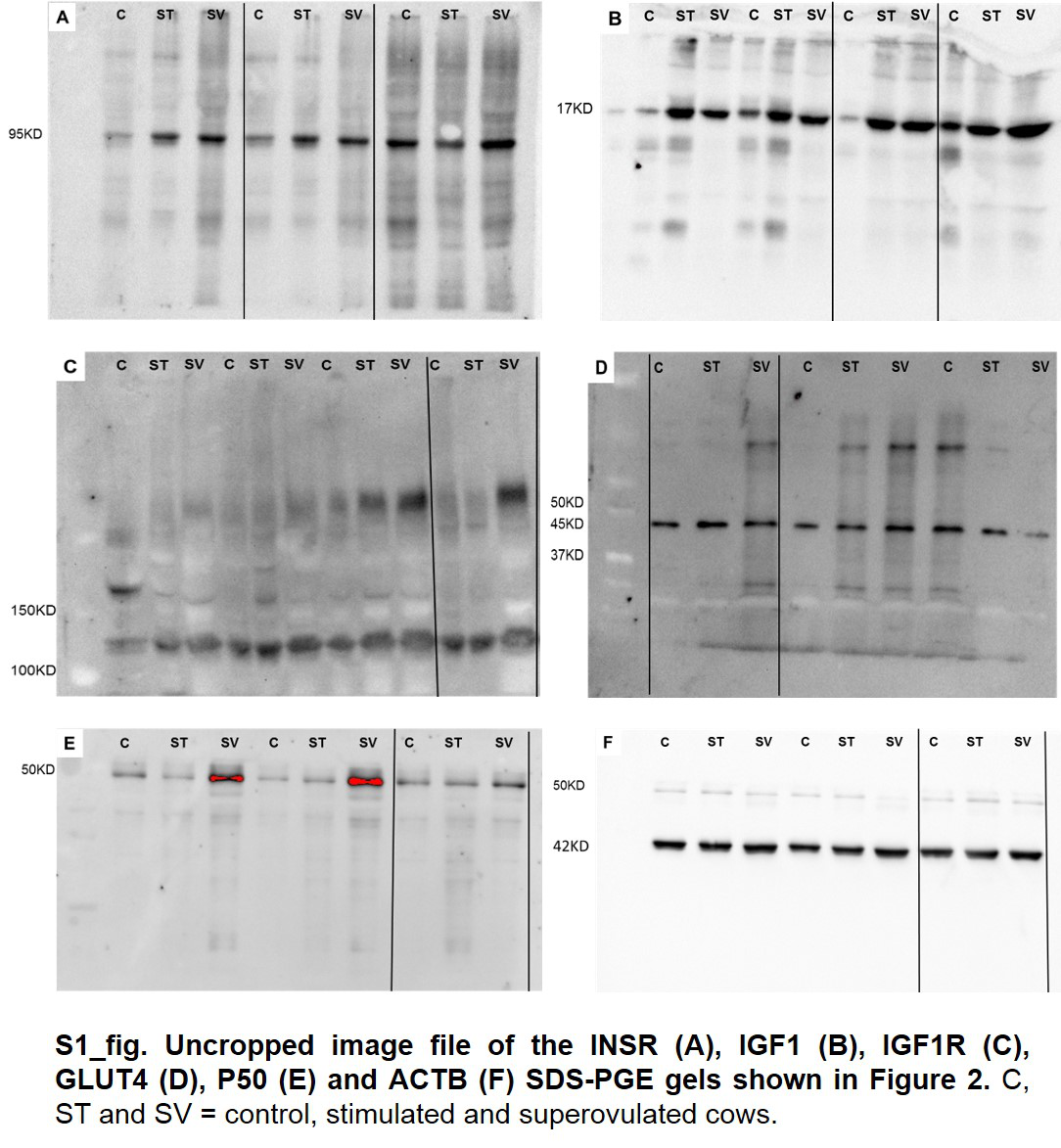

Supplement: S1 Fig — C, ST and SV = control, stimulated and superovulated cows. (TIFF) [file pone.0164089.s001.tiff]

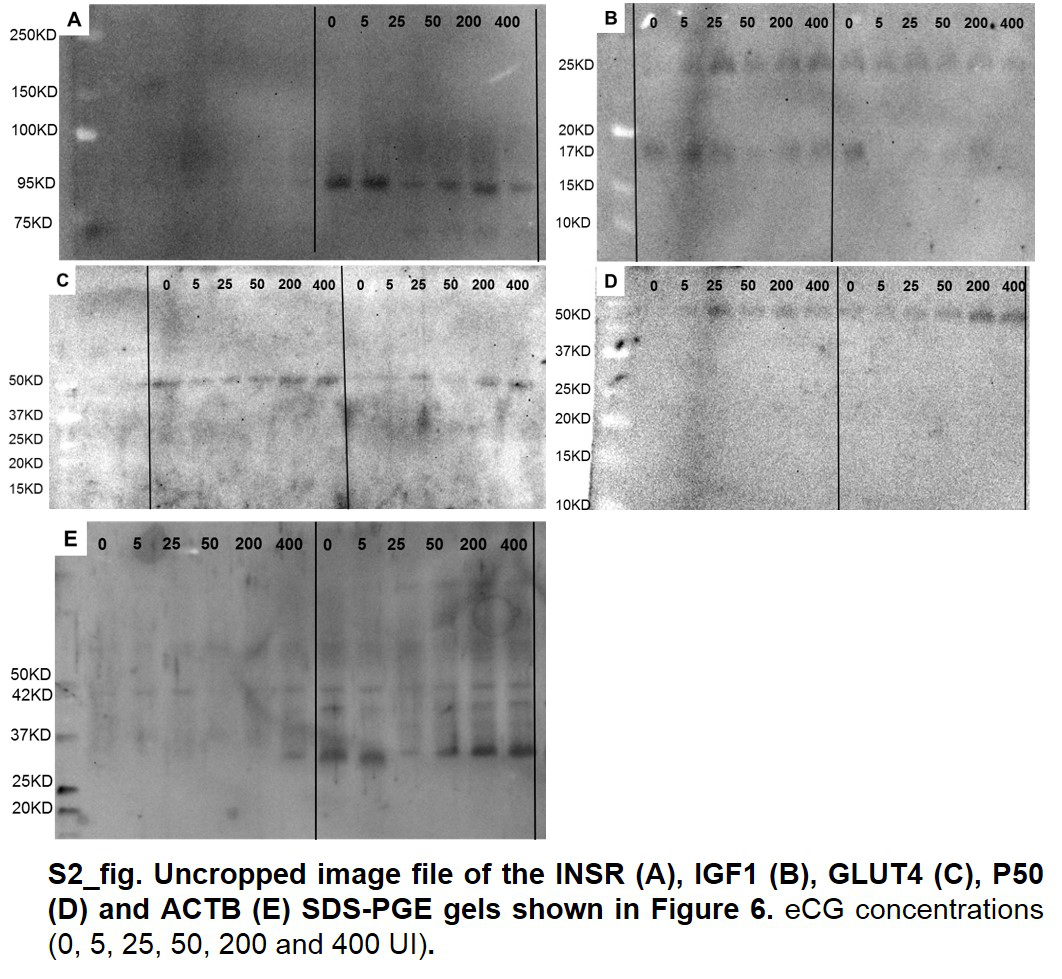

Supplement: S2 Fig — eCG concentrations (0, 5, 25, 50, 200 and 400 UI). (TIFF) [file pone.0164089.s002.tiff]

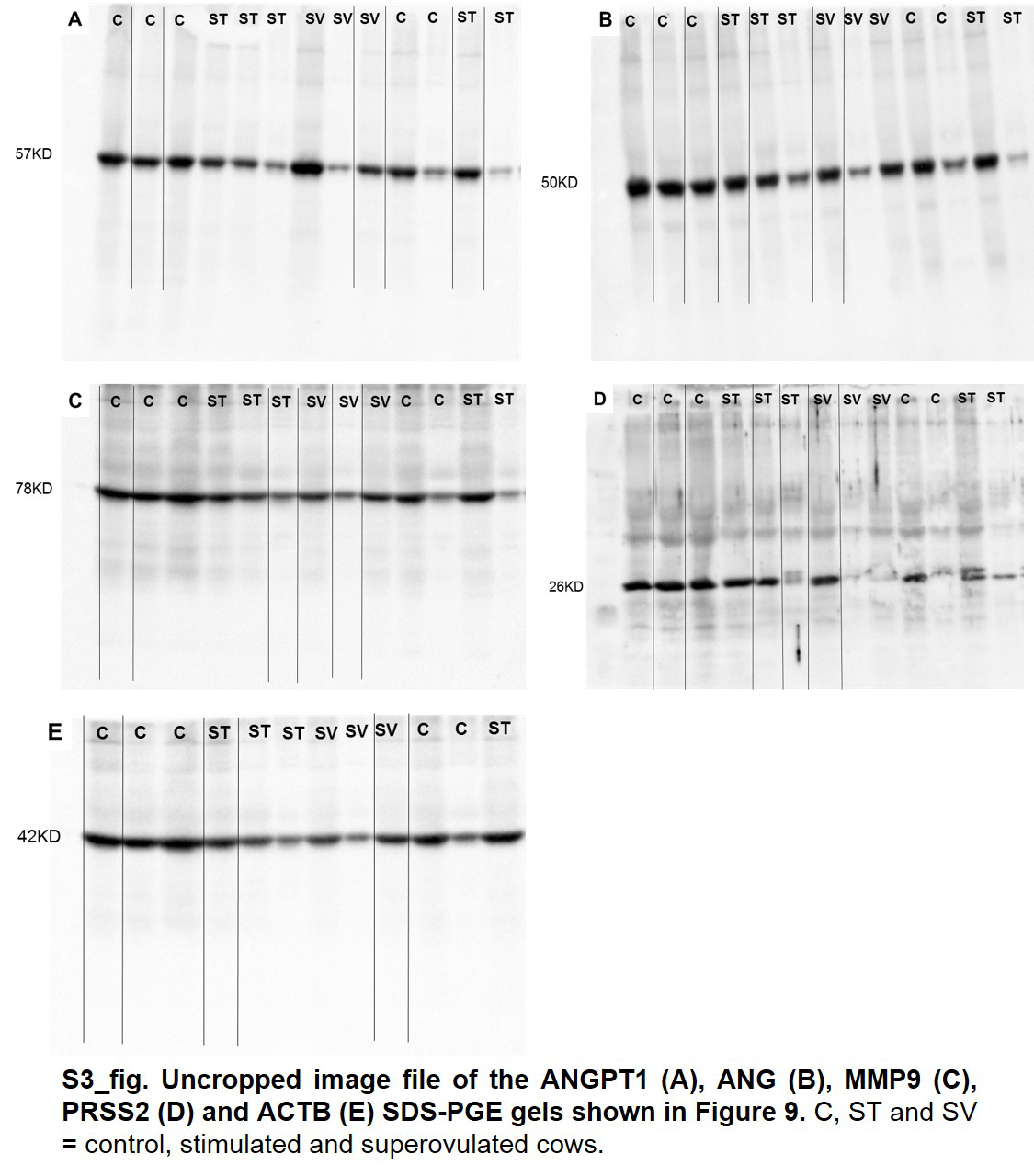

Supplement: S3 Fig — C, ST and SV = control, stimulated and superovulated cows. (TIFF) [file pone.0164089.s003.tiff]

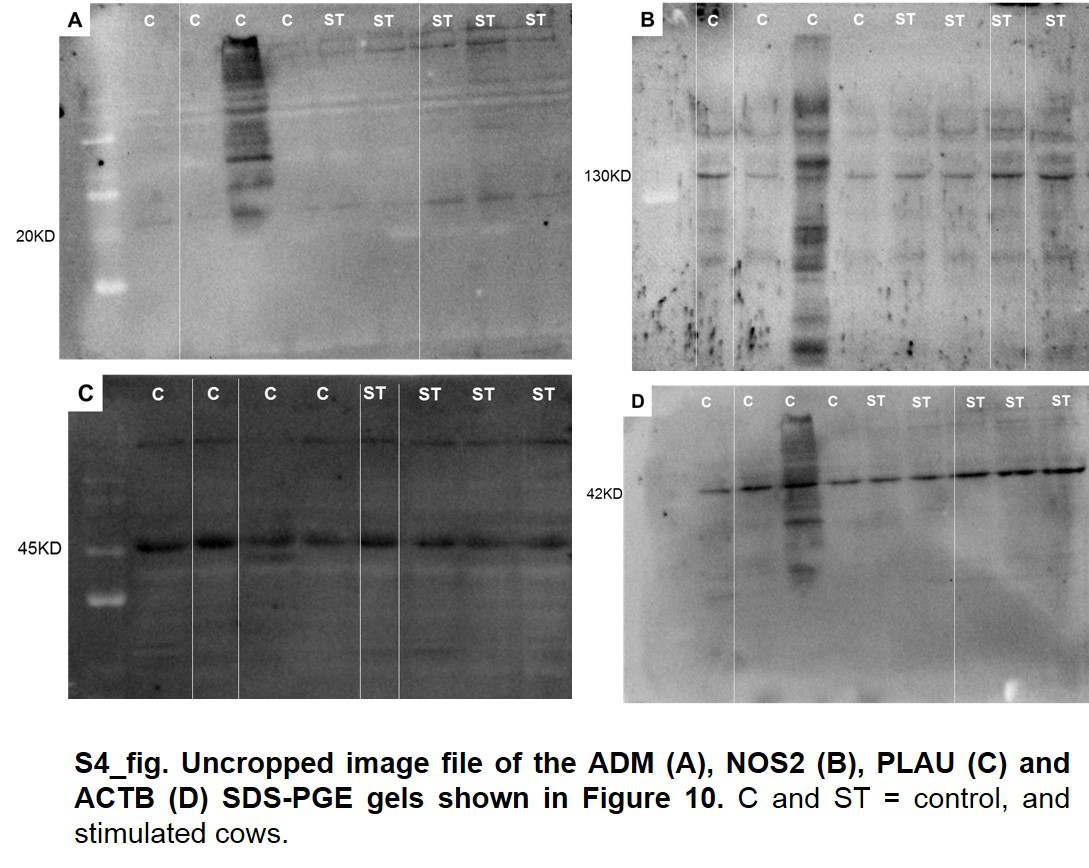

Supplement: S4 Fig — C and ST = control and stimulated cows. (TIFF) [file pone.0164089.s004.tiff]
